# Development of Deep Belief Network for Tool Faults Recognition

**DOI:** 10.3390/s23041872

**Published:** 2023-02-07

**Authors:** Archana P. Kale, Revati M. Wahul, Abhishek D. Patange, Rohan Soman, Wieslaw Ostachowicz

**Affiliations:** 1 Department of Computer Engineering, Modern Education Society’s College of Engineering (MESCOE), Pune 411001, India; 2 Department of Mechanical Engineering, COEP Technological University, Pune 411005, India; 3 Institute of Fluid Flow Machinery, Polish Academy of Sciences, Fiszera 14, 80-231 Gdansk, Poland

**Keywords:** deep belief network, tool faults recognition, face milling, fault diagnosis

## Abstract

The controlled interaction of work material and cutting tool is responsible for the precise outcome of machining activity. Any deviation in cutting parameters such as speed, feed, and depth of cut causes a disturbance to the machining. This leads to the deterioration of a cutting edge and unfinished work material. Recognition and description of tool failure are essential and must be addressed using intelligent techniques. Deep learning is an efficient method that assists in dealing with a large amount of dynamic data. The manufacturing industry generates momentous information every day and has enormous scope for data analysis. Most intelligent systems have been applied toward the prediction of tool conditions; however, they must be explored for descriptive analytics for on-board pattern recognition. In an attempt to recognize the variation in milling operation leading to tool faults, the development of a Deep Belief Network (DBN) is presented. The network intends to classify in total six tool conditions (one healthy and five faulty) through image-based vibration signals acquired in real time. The model was designed, trained, tested, and validated through datasets collected considering diverse input parameters.

## 1. Introduction

Multi-point cutting tools are used for various milling applications such as groove milling, gear machining, shoulder milling, profile milling, face milling, chamfer milling, parting off, holes and cavities/pocketing, etc. [1]. Milling can be further extended to machining operations, drilling, reaming, and broaching by a cutter that rotates in a spindle placed on the turret frame. Work is situated on the table [2]. Conventional machining uses human control of the tools and/or parts to remove the material. Conventional milling tools come in three types: vertical, horizontal, and universal. Conventional tools require skilled operators and are helpful for machining tool room parts or carrying out customized jobs. A Computer Numerical Control (CNC) milling center employs a computer and computer programs to move the cutting tools and/or parts to perform the material removal. Today, conventional machines are being replaced with CNC Vertical Milling Centers (VMCs) due to a lack of skills for high-precision operations [3]. In the manufacturing industry, state of the art technologies are used to ensure that these operations are carried out with as much efficiency as possible [4]. In milling operations, the material is removed when the workpiece is fed against a spinning multi-point cutting tool, and its geometry is essential given the operation dynamics considered [5]. Milling cutters operate using higher feeds possessing a minor entry angle facilitating improved Material Removal Rates (MRRs) owing to gradual entry during the cut. This assists in the protection of the cutting tip due to a decrease in radial pressure and is thus used for machining milling steel and cast iron. A significant benefit from it is achieving high MRRs owing to more extensive DOCs and offering very light cutting, thus producing quality chips [6].

Milling is essentially an interrupted process as it involves a multi-edged cutter that causes the temperature at the cutting edge to fluctuate [7]. However, vibration is considered to be very sensitive to tool conditions. In machining, the tool, tool holder, and spindle vibrate at a controlled natural frequency. The assembly simultaneously vibrates at more than one such natural frequency owing to intermittent cutting [8]. The vibration exerts a wave on the machined surface at the tool edge. The waviness causes a variable load to be experienced by the next cutting edge. After this, the unknown factors disturb the vibration already in place, worsening it [9]. Excessive vibration may be generated during the machining process. The excessive vibrations affect the total material removal rate and, consequently, the machining time [10]. They result in an unacceptable machining finish, and may or may not hold dimensions. For cutting tools, there could be wear or tip failure. Various factors cause this vibration, concerning job material, geometry, and how it is held in the chuck/fixture-excessive overhang, wrong tool material and tool geometry, wrong cutting speed, depth of cut, and feed rates [11]. The reasons could be because of a single factor or a combination. The vibrations can be reduced by balancing rotatory elements, decreasing the clearance in guide-ways, securing support for the job, the proper relief in sharp tools, good cutting velocity for the cutter and workpiece, using the proper coolant/cutting lubricant, cutting at higher speeds with smaller cutters or using solid carbide cutters with randomized flutes, etc. [12].

A brief review is presented here and incorporates research on various methodologies used for condition monitoring, considering sensors, signal processing, and classification techniques. For these operations to work efficiently, it is necessary to ensure that the tool’s condition is normal (healthy) [13]. With this motivation, the concept of developing a Tool Condition Monitoring (TCM) system has evolved. A significant goal of TCM is to recognize the deterioration of the cutting edge, which subsequently improves the value of the job [14,15]. TCM generally aims at monitoring the behavior of the cutting tool in terms of machining datasets collected through various sensors such as microphones [16], dynamometers [17], accelerometers [18], etc.

Ozel and Nadgir [19] used neural networks for fault diagnosis and flank wear was analyzed considering multiple machining factors. Forces acting on a cutting tool pertaining to a typical cutting condition were measured using a piezoelectric-based dynamometer. Fang et al. [20] transformed signals with the combination of discrete wavelet transform and fast Fourier transform for analyzing the vibrations corresponding to the wear of the tool edge. In addition to this, principal component analysis was employed to analyze cutting force variation during the machining. Elangovan et al. [21] advocated an intelligent data-driven method for examining tool condition based on vibrations produced during interaction of cutting tool and workpiece. These vibrations were then analyzed using statistical features as well as histogram plots followed by categorization of tool faults using a Bayesian approach. In order to estimate remaining useful life, Satishkumar and Sugumaran [22] demonstrated the suitability of a nested dichotomy algorithm trained through statistical features of vibration signal. Signal transformation and its representation play a vital role in signal processing. For vibration-based analysis, time, frequency, and time–frequency domains are usually considered [23]. White light interferometry was utilized for measurement of crater wear on a number of replaceable cutting inserts [24]. Abu-Zahra and Yu [25] suggested applicability of discrete wave transform for measurement of deterioration of a cutting edge of replaceable cutting tool tips for ultrasound waves. Further, a multi-layer perceptron (MLP) was trained and correlations were found with the help of wavelet packets. The comparative study of neural nets and hidden Markov models was provided by Scheffer et al. [26] while developing a regression model for prediction of tool condition. Liu and Jolley [27] obtained an index by employing three axis cutting forces. The index was analyzed using the counter-propagative neural net. Recently, Patange et al. [28] suggested an ML approach to diagnose the condition of a milling cutter. Relevant features were selected from a decision tree and for several tool conditions and classified using a random forest tree algorithm. The use of the Bayes net, naïve Bayes, and tree family classifiers for classification between healthy and defective tools has been demonstrated [29,30,31]. Statistical features from the vibrational data were extracted and dimensionality reduction was undertaken using the J48 classifier [32,33].

**Figure 1:**
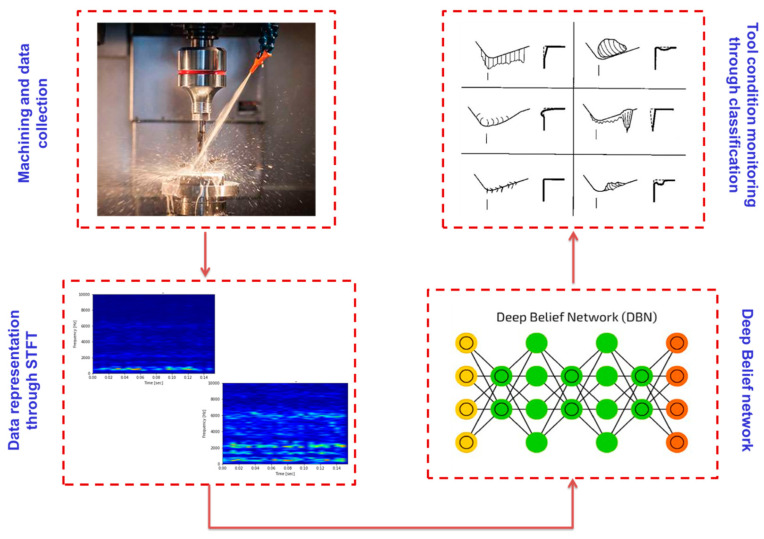
Step-wise organization of the current investigation.

To retain the quality of the cutting tool throughout its lifecycle, the deep learning approach is thus advocated to make the model robust for noisy environments and cross-domain conditions. It creates new learning to monitor cutting tools. The current investigation exclusively describes a method to identify different tool wear states using a deep learning-based scheme. The model uses vibration signals of the spindle frame to detect the deterioration of the cutting edge from a rigorous experimental investigation. The DBN model is designed to classify different wear states instead of a simple tool that is worn and not worn through a binary classification, as illustrated in Figure 1. This investigation supports the ongoing revolution of industry that demands the adoption of self-monitoring of machines.

## 2. Experiments and Signal Acquisition

**Figure 2:**
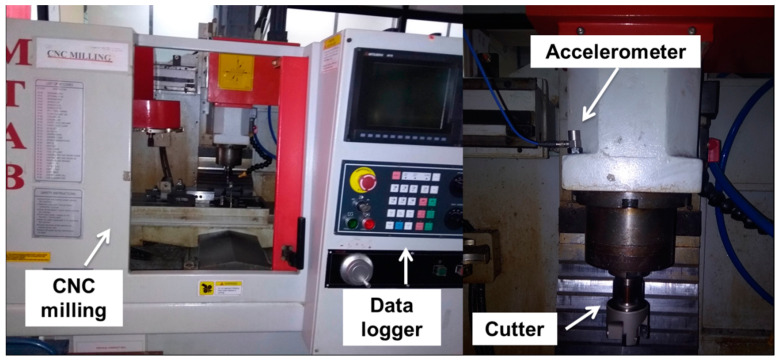
The schematic of the experimentation arrangement.

In order to undertake real-time signal acquisition, the machining experiments were performed on a CNC milling tool. The investigation based on Bayes algorithms and metaheuristic algorithm driven SVM was carried out using same experimental setup with numeric vibration data [29,30]. The setup is shown in Figure 2 which consists of a machine tool, a cutting tool mounted inside the spindle, a work material, a sensor—piezoelectric accelerometer—and a data logger. The job material was mild steel which was face milled with a cutter consisting of four tips and depth of cut of 0.2 mm, speed of 1000 revolutions per minute, and feed of 48 mm/min were selected. The machining was carried out considering six tool conditions in total. The single-axial piezoelectric accelerometer was selected. Vibration signatures developed throughout machining pass on to the spindle holder sensitively and thus when recording tool faults, any variation in spindle motion can be detected if the sensor is located near to it. For vertical milling, the component of vibration in the Z-direction is the most sensitive to tool faults rather than X and Y, so the sensor was directly attached to a spindle block in the vertical direction. A data logger was engaged to collect a time-domain response. With respect to the formula given by Nyquist, a sampling was undertaken at 24 kHz. Here, in total 240 samples were collected which means each tool label is represented by 40 samples. For testing robustness of the trained model, a separate dataset was acquired independently and served in the model as a blind dataset. While acquiring this dataset, different CNC trainers of the same model were utilized. The experimentation was carried out considering speed, feed, and depth of cut as mentioned earlier till the cutting tool failed.

## 3. Signal Representation and Data Preparation through STFT

**Figure 3:**
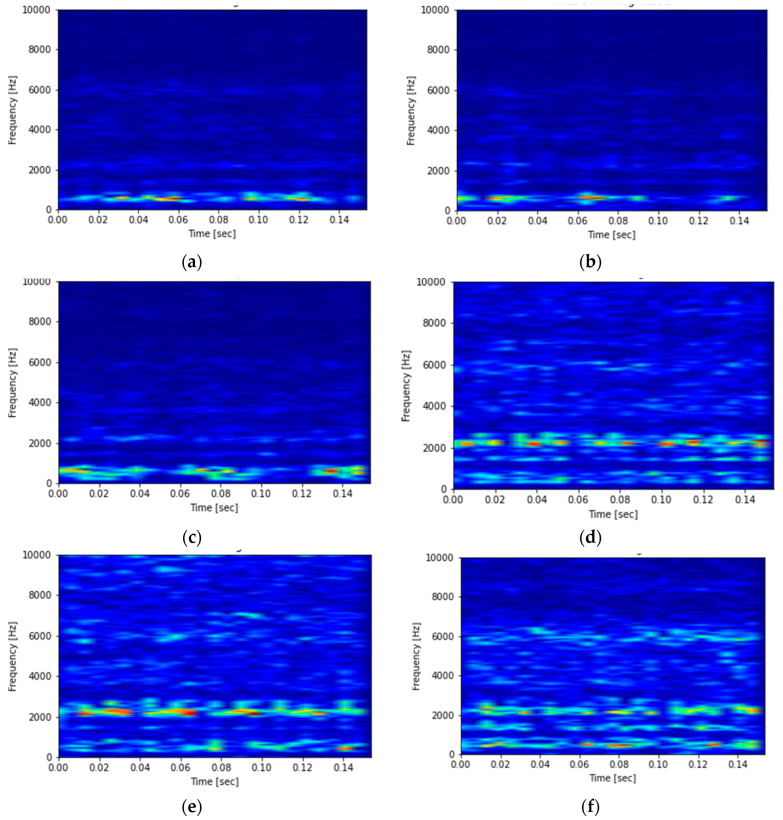
STFT Spectrogram representing various tool conditions.

Variation in vibration levels is acquired primarily in the time domain directly from the sensors used to measure its acceleration, velocity, and displacement by connecting them to the vibration DAQs. For very basic investigation, a simple vibration meter shows the intensity of vibrations with a linear measurement dependent on time such as mm/sec. Some spring mass mechanisms are used for conversion of change in motion into an electrical signal. When dynamic and random vibrations are considered, there is need for some systematic signal processing using sophisticated techniques. Conventionally, a vibration consultant or maintenance engineer used to observe vibration patterns and a decision would be undertaken regarding fault diagnosis with their experience. These specialists used to recognize signatures through their knowledge, but the assessment was very subjective. A very well-known method of transforming time-domain signal into frequency-domain signal is Fast Fourier Transform (FFT). Basically, Fourier Transform (FT) is used when there is need for non-periodic signals to be transformed into infinite harmonics of distinct frequencies and corresponding amplitudes. Usually, FT employs signal processing to recognize either time or frequency domains. However, important information about vibration patterns from the time domain disappears as FT converts non-periodic signals to infinite harmonics of distinct frequencies and corresponding amplitudes. To tackle this problem, the adoption of Short-Time Fourier Transform (STFT) is desired because it retains frequency components of local time intervals of fixed duration [34,35]. However, vibrations with fast transients (i.e., high-frequency content for a short duration) need to be analyzed using Wavelet Transform (WT). The duration of this ‘short time’ in Fourier transform serves as an important factor in comparison of STFT and wavelet as it changes in wavelet transform, thus the time window of the signal is adaptive [36]. As the frequency of signal increases, the duration of the window shortens and at the same time the frequency spectrum expands. For low-frequency signals, the window extends in time and shortens in frequency. The duration of the window for the STFT is fixed in both time and frequency. Different shades in an STFT spectrogram are an indicator of the uneven resolution of time–frequency. The window type and length determine the energy blurriness across time and frequency and time and frequency resolution in the STFT spectrogram [37]. For a larger window owing to greater blurring across time, there is a decrement in energy blurring across the range of frequencies. Conversely, a narrower window decreases blurring across time owing to greater blurring across the range of frequencies. Figure 3a–f depict the vibration signal in the form of an STFT spectrogram for six tool conditions in total (one healthy and five faulty).

The machining time was 20 s in total for each tool condition. The disturbance in the STFT spectrogram for healthy tools is less as compared to the faulty ones which shows the coarse time–frequency resolution. For operations Figure 3b–e, the characteristics of vibration are varied in terms of fogginess in the STFT spectrogram as compared to healthy tools as shown in Figure 3a. For operation Figure 3f, the characteristics of vibration are completely foggy in the STFT spectrogram as compared to other tool conditions. The STFT spectrogram graph portrays the behavior of signals conforming to the sudden effect of faults. This kind of distinctive nature of signals is essential for the algorithm to understand. Various tool conditions consisted in operations imitating the distinct variation in real-time signals captured. In order to recognize these conditions, a deep belief network is designed, trained, and tested, and is explained in the next section.

## 4. Deep Belief Network (DBN)

**Figure 4:**
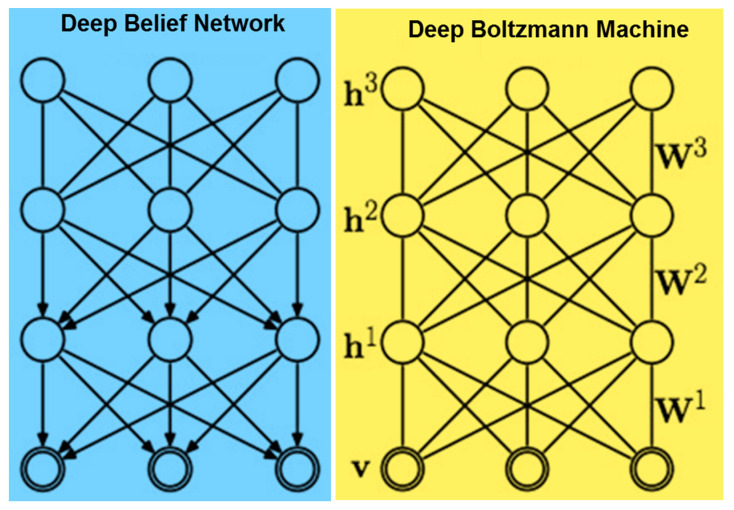
Mechanism of Deep Belief Network and Deep Boltzmann Machine.

**Figure 5:**
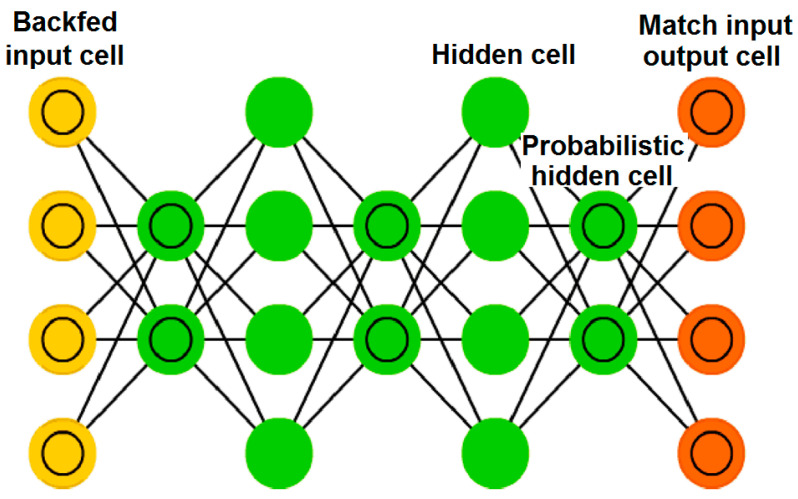
Architecture of Deep Belief Network.

The deep learning approach is used for stacking shallow architectures and achieving robust results due to increasing depth of the model. The deep belief networks are composed of Restricted Boltzmann Machines (RBMs). In deep networks, in place of using a single layer of RBM, they are stacked on top of each other, called a DBN as shown in Figure 4 [38]. The term ‘belief’ in mathematics might create confusion. Commonly, a belief is something that one has faith in, such as a deity, or science, or trusting astrology, etc. However, in DBNs, the term ‘belief’ expresses the knowledge that one may have a flawed subjective conclusion about the objective indication [39]. A DBN is also a type of deep learning-based deterministic neural net and chooses weights in a smart way according to the activation functions as shown in Figure 5.

The primary motivation behind the use of deep learning is to gain a hierarchical representation of the data for a particular problem, such as classification, regression, clustering, and ranking. Of course, a DBN is one such approach to the implementation of deep learning architectures. However, at the time of its development, it was challenging to make it work. In the case of the Bayesian network, links project probabilistic dependence, however, in a neural net, there is a neuron association [40]. The layers of a DBN learn a hierarchical representation and are driven by restricted Boltzmann machines stacked on top of one another [41]. To put it in simple words, a restricted Boltzmann machine is found at every level of the DBN as per its characteristic. Usually, an unsupervised approach is used to train a DBN level by level. Once the first RDM is trained, another one is stacked on top, and the procedure is iterated. The assembly of RBMs to design a DBN is the same as assembling auto-encoders to train a stacked auto-encoder. Since DBNs are generative probabilistic models, they are designed by generation of the latent representation at the present level by consuming the one from the preceding level as its input, making them competent for dynamic data distributions [42].

## 5. Design of DBN

The design of a deep belief network is presented in this section which has a total of 240 inputs from each tool condition and has to recognize 6 classes. The network has to learn from the STFT spectrogram of tool conditions and would classify the recognized pattern into one of the six classes. The DBN assists in prognostic-based categorization activities owing to its capability of feature engineering, including detection and extraction [43]. The DBN classifier comprises a topmost layer association memory, an RBM classifier that allows training of the top level to produce labels of class conforming to the input STFT images. The classification through the DBN requires the tags of observation at the time of training the topmost layer, so it carried out the training of the bottommost layer first, propagates the images via the trained RBM, and later uses transformed images as a training dataset for the training of subsequent RBMs. This continues till the whole dataset propagates via the latest trained RBMs, and the tags are concatenated with transformed images and employed for training of the topmost layer association memory [44]. Learning is accomplished via both approaches, i.e., unsupervised and supervised. The unsupervised process starts when the dataset propagates from the bottommost to the topmost layer and vice versa [45,46]. The decisive step in the training of the DBN is hyperparameter tuning. The hyperparameters are the count of epochs, batch sizing, dropout, count of hidden layers, and learning rate. They play a vital role as they control the learning and training procedures, which significantly influence the capabilities of the architecture under consideration. The values chosen for the training of the DBN model are shown in Table 1.

In total, 240 STFT spectrograms representing 6 classes, i.e., 40 STFT spectrograms per class, were considered to be the complete dataset. This STFT spectrogram dataset was divided into a training dataset and testing dataset with a split of 80/20, respectively. The validation was undertaken considering separate blind data. Along with this, the model was trained using a 10-fold cross-validation approach.

## 6. Results and Discussion

The recognition and classification are the decisive steps in DL-based predictive analytics. They involve training and testing algorithms with the set of STFT spectrograms. Designing or training is defined as a process of learning from the labeled dataset. The training problem can be either unsupervised or supervised. Unsupervised training is used to classify unlabeled datasets and clustering is carried out. In the current investigation, the condition for which vibration data was collected is known. Thus, the supervised approach is employed to design and train the classifiers. On the other hand, testing is defined as a process of inspecting a trained model of the classifier with unseen instances with labels. However, testing is considered for checking the trained model. First, the models are constructed considering the k-fold cross-validation mode. The cross-validation test mode with k = 10, i.e., ten folds of equal size, was created amongst a complete dataset of 240 samples. A single fold out of ten folds was reserved as the authenticating data to test the classifier, and the leftover nine folds were employed for training. The procedure has to be repeated for ten intervals such that every fold is explored as test data one by one. The averaged estimation was then determined from the ‘k’ results. The classification and evaluated are represented with the help of the confusion matrix. The name ‘confusion’ reflects which sort of confusion occurs in the prediction of a class. The shape of a confusion matrix is a square matrix specified by ‘N × N’, where ‘N’ is rows and columns which are decided with respect to the number of classes involved in training. In this study, a multi-class ‘6 × 6′ matrix is formed using the 6 classes.

The samples placed in each row indicate the true class and samples placed in each column indicate the predicted class. All correctly classified instances are placed in the major diagonal (highlighted in bold), and values outside the diagonal represent incorrectly classified instances.

**Figure 6:**
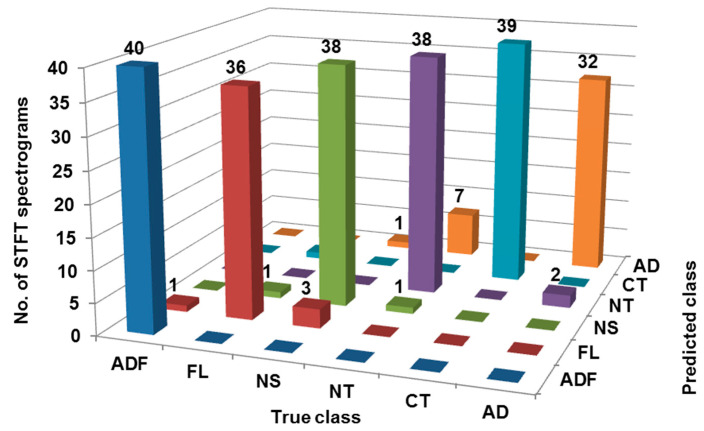
Confusion matrix considering cross-validation mode (ADF: All defect free (healthy), FL: Flank wear, NS: Nose wear, NT: Notch, CT: Crater, AD: All defective).

The confusion matrix from Figure 6 shows that for class ‘ADF’ all 40 STFT spectrograms are correctly classified thus there is no misclassification. For class ‘FL’, only 36 STFT spectrograms are correctly classified whereas 1 and 3 were misclassified as ‘ADF’ and ‘NS’, respectively. For class ‘NS’, only 38 STFT spectrograms are correctly classified whereas an STFT spectrogram was misclassified as ‘FL’ and ‘NT’ each. Further, for class ‘NT’, only 38 STFT spectrograms are correctly classified whereas 2 STFT spectrograms were misclassified as ‘AD’. For class ‘CT’, only 39 STFT spectrograms are correctly classified whereas 1 STFT spectrogram was misclassified as ‘FL’. Lastly, for class ‘AD’, only 32 STFT spectrograms are correctly classified whereas 7 and 1 STFT spectrograms were misclassified as ‘NS’ and ‘NT’. Therefore, as shown in Table 2, 223 out of 240 STFT spectrograms are correctly classified, hence the classification accuracy is 92.91%. Table 3 shows various performance indicators of classification considering cross-validation mode such as ROC, MCC, F-measure, Precision, TP rate, Recall, FP rate, PRC, etc.

**Figure 7:**
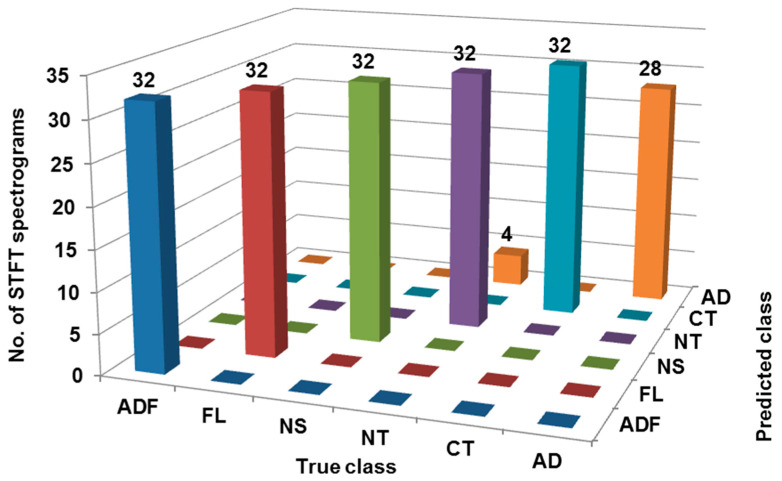
Confusion matrix considering training dataset.

The confusion matrix was generated using the DBN classifier considering the training dataset as presented in Figure 7. For class ‘ADF’, all 32 STFT spectrograms are correctly classified, thus there is no misclassification. For class ‘FL’, all 32 STFT spectrograms are correctly classified, thus there is no misclassification. For class ‘NS’, all 32 STFT spectrograms are correctly classified, thus there is no misclassification. Further, for class ‘NT’, all 32 STFT spectrograms are correctly classified, thus there is no misclassification. For class ‘CT’, all 32 STFT spectrograms are correctly classified, thus there is no misclassification. Lastly, for class ‘AD’, only 28 STFT spectrograms are correctly classified whereas 4 STFT spectrograms were misclassified as ‘NT’. Therefore, as shown in Table 4, 188 out of 192 STFT spectrograms are correctly classified, hence the classification accuracy is 97.91%. Table 5 shows various performance indicators of classification considering the training dataset such as ROC, MCC, F-measure, Precision, TP rate, Recall, FP rate, PRC, etc.

**Figure 8:**
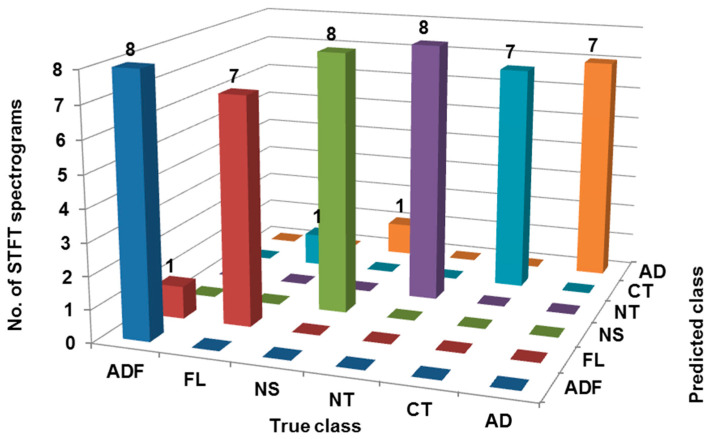
Confusion matrix considering test dataset.

The confusion matrix was generated using the DBN classifier considering the test dataset as presented in Figure 8.

For class ‘ADF’, all eight STFT spectrograms are correctly classified, thus there is no misclassification. For class ‘FL’, only seven STFT spectrograms are correctly classified whereas one was misclassified as ‘ADF’. For class ‘NS’, all eight STFT spectrograms are correctly classified, thus there is no misclassification. Further, for class ‘NT’, all eight STFT spectrograms are correctly classified, thus there is no misclassification. For class ‘CT’, only seven STFT spectrograms are correctly classified whereas one STFT spectrogram was misclassified as ‘FL’. Lastly, for class ‘AD’, only seven STFT spectrograms are correctly classified whereas one STFT spectrogram was misclassified as ‘NS’. Therefore, as shown in Table 6, 45 out of 48 STFT spectrograms are correctly classified, hence the classification accuracy is 93.75%. Table 7 shows various performance indicators of classification considering the test dataset such as ROC, MCC, F-measure, Precision, TP rate, Recall, FP rate, PRC, etc.

**Figure 9:**
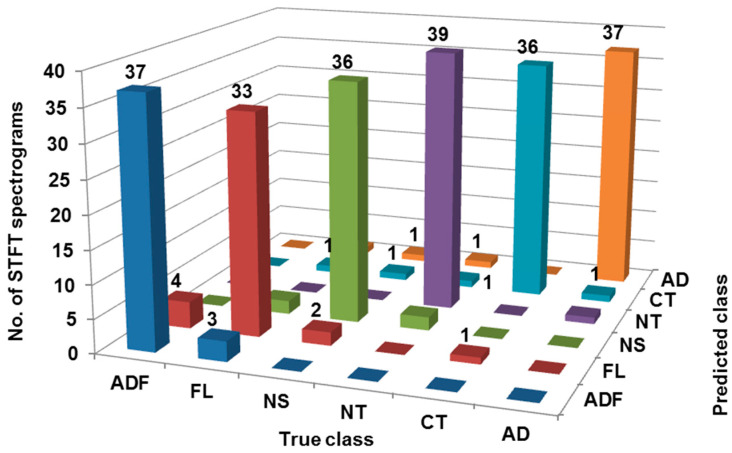
Confusion matrix considering blind dataset.

The confusion matrix from Figure 9 shows that for class ‘ADF’, only 37 STFT spectrograms are correctly classified whereas 3 were misclassified as ‘FL’. For class ‘FL’, only 33 STFT spectrograms are correctly classified whereas 4, 2, and 1 were misclassified as ‘ADF’ ‘NS’, and ‘CT’, respectively.

For class ‘NS’, only 36 STFT spectrograms are correctly classified whereas 2 STFT spectrograms were misclassified as ‘FL’ and ‘NT’ each. Further, for class ‘NT’, only 39 STFT spectrograms are correctly classified whereas 1 STFT spectrogram was misclassified as ‘AD’. For class ‘CT’, only 36 STFT spectrograms are correctly classified whereas 1 STFT spectrogram was misclassified as ‘FL’, ‘NS’, and ‘AD’ each. Lastly, for class ‘AD’, only 37 STFT spectrograms are correctly classified whereas 1 STFT spectrogram was misclassified as ‘FL’, ‘NS’ and ‘NT’ each. Therefore, 218 out of 240 STFT spectrograms are correctly classified, hence the classification accuracy is 90.83% as shown in Table 8. Table 9 shows various performance indicators of classification considering the blind dataset.

## 7. Conclusions

A deep belief network was successfully developed in this paper and is able to recognize the variation in milling operation leading to tool faults. The network is capable of classifying six tool conditions in total (one healthy and five faulty) through image-based vibration signals represented using STFT spectrograms. The model was effectively designed, appropriately trained, aptly tested, and properly validated. Two hundred and twenty-three out of two hundred and forty STFT spectrograms were correctly classified, hence the classification accuracy is 92.91% considering cross-validation mode. For classification considering the training dataset, 188 out of 192 STFT spectrograms are correctly classified, hence the classification accuracy is 97.91%. Forty-five out of forty-eight STFT spectrograms are correctly classified, hence the classification accuracy is 93.75% considering the test dataset. For classification considering the blind dataset, 218 out of 240 STFT spectrograms are correctly classified, hence the classification accuracy is 90.83%. This makes application of STFT spectrograms based on vibration signals suitable for tool faults diagnosis. This trained model of classification shall be considered for data from other machines and is possibly to be encouraged for further deployment for cross-domain data distribution. Further, the investigation could be extended considering fusion of vibration and sound signal using adversarial training, domain adaptation, transfer learning, etc.

## Figures and Tables

**Table 1:** Hyperparameters chosen.

Parameter	Epochs	Batch	Learning Rate	Dropout	Number of Nodes
**RBM**	60	256	0.05	NA	500
**DBN**	400	NA	0.1	0.2	NA

**Table 2:** Classification results summary considering cross-validation mode.

Classification Report	
Total number of samples utilized for training	240
Number of samples accurately classified by trained model	223
Percentage accuracy of classification	92.91
Number of samples wrongly classified by trained model	17
Percentage misclassification	7.083

**Table 3:** Performance indicators of classification considering cross-validation mode.

ROC	MCC	F-Measure	Precision	TP Rate	Recall	FP Rate	PRC	Tool State
100	98.5	98.8	97.6	100	100	0.5	100	ADF
99.5	90.9	92.3	94.7	90.0	90.0	1.0	96.7	FL
99.1	91.2	92.7	90.5	95.0	95.0	2.0	97.5	NS
98.9	86.2	88.4	82.6	95.0	95.0	4.0	95.0	NT
100	98.5	98.7	100	97.5	97.5	0.0	100	CT
98.9	84.4	86.5	94.1	80.0	80.0	1.0	94.3	AD

**Table 4:** Classification results summary considering training dataset.

Classification	
Total number of samples utilized for training	192
Number of samples accurately classified by trained model	188
Percentage accuracy of classification	97.91
Number of samples wrongly classified by trained model	4
Percentage misclassification	2.083

**Table 5:** Performance indicators of classification considering training dataset.

ROC	MCC	F-Measure	Precision	TP Rate	Recall	FP Rate	PRC	Tool State
100	100	100	100	100	100	00	100	**ADF**
100	100	100	100	100	100	00	100	**FL**
100	100	100	100	100	100	00	100	**NS**
99.5	93.1	94.1	88.9	100	100	2.5	96.9	**NT**
100	100	100	100	100	100	00	100	**CT**
99.5	92.4	93.3	100	87.5	87.5	00	97.5	**AD**

**Table 6:** Classification results summary considering test dataset.

Classification	
Total number of samples utilized for training	48
Number of samples accurately classified by trained model	45
Percentage accuracy of classification	93.75
Number of samples wrongly classified by trained model	3
Percentage misclassification	6.25

**Table 7:** Performance indicators of classification considering test dataset.

ROC	MCC	F-Measure	Precision	TP Rate	Recall	FP Rate	PRC	Tool State
100	93.1	94.1	88.9	100	100	2.5	100	**ADF**
99.8	85.0	87.5	87.5	97.5	97.5	2.5	98.6	**FL**
100	93.1	94.1	88.9	100	100	2.5	100	**NS**
100	100	100	100	100	100	0.0	100	**NT**
99.8	92.4	93.3	100	87.5	87.5	0.0	98.6	**CT**
99.8	92.4	93.3	100	87.5	87.5	0.0	89.9	**AD**

**Table 8:** Classification results summary considering blind dataset.

Classification	
Total number of samples utilized for training	240
Number of samples accurately classified by trained model	218
Percentage accuracy of classification	90.83
Number of samples wrongly classified by trained model	22
Percentage misclassification	9.17

**Table 9:** Performance indicators of classification considering blind dataset.

ROC	MCC	F-Measure	Precision	TP Rate	Recall	FP Rate	PRC	Tool State
99.7	89.6	91.4	90.2	92.5	92.5	2.0	98.4	**ADF**
98.7	79.0	82.5	82.5	82.5	82.5	3.5	94.2	**FL**
99.3	88.0	90.0	90.0	90.0	90.0	2.0	97.2	**NS**
99.4	92.8	94.0	90.7	97.5	97.5	2.0	95.7	**NT**
99.8	92.4	93.5	97.3	90.0	90.0	0.5	99.2	**CT**
99.8	92.4	93.7	94.9	92.5	92.5	1.0	99.0	**AD**

## Data Availability

The data presented in this study are available on request from the authors. The data are not publicly available as they are part of ongoing research work.
